# 3D SSY Estimate of EPFM Constraint Parameter under Biaxial Loading for Sensor Structure Design†

**DOI:** 10.3390/s19030735

**Published:** 2019-02-12

**Authors:** Ping Ding, Xin Wang

**Affiliations:** 1 National Research Base of Intelligent Manufacturing Service, Chongqing Technology and Business University, Chongqing 400067, China; 2 Department of Mechanical and Aerospace Engineering, Carleton University, Ottawa, ON K1S 5B6, Canada

**Keywords:** sensor design, sensor structure analysis, two parameter approach, constraint parameter, small scale yielding, *T*-stress, estimate method, three-dimensional, biaxial loading

## Abstract

Conventional sensor structure design and related fracture mechanics analysis are based on the single *J*-integral parameter approach of elastic-plastic fracture mechanics (EPFM). Under low crack constraint cases, the EPFM one-parameter approach generally gives a stress overestimate, which results in a great cost waste of labor and sensor components. The *J-A* two-parameter approach overcomes this limitation. To enable the extensive application of the *J-A* approach on theoretical research and sensor engineering problem, under small scale yielding (SSY) conditions, the authors developed an estimate method to conveniently and quickly obtain the constraint (second) parameter *A* values directly from *T*-stress. Practical engineering application of sensor structure analysis and design focuses on three-dimensional (3D) structures with biaxial external loading, while the estimate method was developed based on two-dimensional (2D) plain strain condition with uniaxial loading. In the current work, the estimate method was successfully extended to a 3D structure with biaxial loading cases, which is appropriate for practical sensor design. The estimate method extension and validation process was implemented through a thin 3D single edge cracked plate (SECP) specimen. The process implementation was completed in two specified planes of 3D SECP along model thickness. A wide range of material and geometrical properties were applied for the extension and validation process, with material hardening exponent value 3, 5 and 10, and crack length ratio 0.1, 0.3 and 0.7.

## 1. Introduction

Fracture mechanics analysis (e.g., [1,2,3]) is significant for engineering structure analysis and design process. In sensor structure analysis and design process, especially supporting bracket design, sensor structure elastic-plastic fracture mechanics (EPFM) analysis is essential to ensure reliability and service life of sensors, and those of prognostics and health management (PHM) systems, accordingly [4,5,6], e.g., the battery management system.

The conventional one-parameter *J*-based approach [7,8] of EPFM usually only works well for cases of sensor structure with high crack constraint. For cases with a low EPFM crack constraint, one-parameter *J*-based approach [7,8] usually does not have enough accuracy to character constraint effect on crack-front stress and strain fields. In such a situation, stress values of sensor structures are usually overestimated by the one-parameter approach. This will result in an inappropriate structure maintenance strategy, and lead to unnecessary component replacement and labor costs, accordingly.

Describing the applied load through the *J*-integral [9] (like that in *J*-based one-parameter approach), several EPFM two-parameter approaches have been developed to overcome the one-parameter approach limitation and obtain a more accurate crack-tip (-front) field description, with a second parameter illustrating the crack constraint effect. Through extending Williams’ *K-T* two-parameter approach [10] of elastic materials to elastic-plastic materials, Betegon and Hancock [11], as well as Al-Ani and Hancock [12], developed the *J-T* two-parameter approach. O’Dowd and Shih [13,14] proposed the *J-Q* two-parameter approach, with a constraint (second) fracture parameter *Q*. With constraint parameter *A*_2_, Yang, et al. [15] proposed the *J-A*_2_ two-parameter approach. Using parameter *A*, an *A*_2_ different normalizing form for constrain description, Nikishkov et al. [16,17] derived an alternative format of *J-A*_2_, *J-A* two-parameter approach.

It has been well known that the two-parameter approaches provide effective characterization of plane strain elastic-plastic crack-tip fields in a variety of structure crack configurations and loading conditions. Current defect assessments and structural integrity procedures used by sensor structure analysis and design have incorporated the constraint effects, see reference [18] for an example.

To apply the EPFM two-parameter approaches on sensor structure analysis and design, the precondition is the value determination of *J*-integral and constraint parameter, *T*-stress, *Q* and *A*_2_ (*A*). As the parameter of the conventional one-parameter approach, *J*-integral values had been well available. Furthermore, as a linear elastic constraint parameter, solutions of *T*-stress have been well established for both numerical and analytical ones, see reference [19] and [20] for examples. Conversely, solutions of elastic-plastic constraint parameters, *Q* and *A*_2_ (*A*), have not been well developed, since they are related to material nonlinearities. 

Theoretically, the *Q* or *A*_2_ (*A*) parameter values could be determined numerically based on finite element analysis (FEA), such as the fitting method proposed by Nikishkov et al. [16,17], which obtains parameter *A* values from FEA results. For a wide range of material, geometric properties and loading, determining the values of *Q* and *A*_2_ (*A*) numerically is quite a time-consuming work. The numerical method is not appropriate for sensor structure analysis and other engineering applications, even extensive theoretical investigation. Estimate (predicting) methods should be developed systematically to obtain the values of constraint parameters *A*_2_ (*A*) and *Q* quickly and conveniently for convenient sensor structure design and other practical engineering applications. 

As mentioned in previous paragraphs, both numerical and analytical solutions of elastic *T*-stress have been well established. There should be a possibility that constraint parameter *A* values could be obtained from *T*-stress directly, provided the relationship between *A* and *T*-stress could be determined. *T*-stress term is an elastic parameter; it is only appropriate for EPFM small scale yielding (SSY) conditions. Therefore, the above mentioned relationship between parameter *A* and *T*-stress should only be valid under the SSY condition of sensor structure EPFM analysis. 

Based on the determined *A-T* relationship, an SSY estimate method for constraint parameter *A* was developed by the authors [21] under two-dimensional (2D) plain strain condition, and was then extended to a three-dimensional (3D) structure with uniaxial external loading [22]. It predicts parameter *A* solutions directly from *T*-stress values conveniently and quickly, to enable the practical engineering application of *T*-stress-based estimate method for parameter *A*.

Biaxial external loadings are experienced by sensor structures and many other engineering components. Thus, they are of more important engineering practical significance than uniaxial loading cases. In the present work, the suggested SSY estimate methodology [21,23] for constraint parameter *A* will be extended to 3D biaxial external loading condition for sensor structure analysis and design as well as other engineering applications through 3D single edge cracked plate (SECP) specimen analysis.

The rest of the paper is organized as follows. In Section 2, related theoretical background will be summarized. Extensive FEAs and parameter *A* numerical solutions will be discussed in Section 3 for thin 3D SECP cracked specimens under biaxial loading conditions with biaxial ratio *λ* = 1.0. In Section 4, 3D *T*-stress-based estimate methodology (SSY method) is extended to biaxial loading conditions for sensor design application, and the extension is validated. Conclusions will be drawn in Section 5. The results in current work will greatly benefit the sensor design process and other engineering applications.

## 2. Theoretical Background

### 2.1. J-T and J-A Two-Parameter Approach

According to Williams’ [10] series solution for 2D crack-tip stress fields, one gets:

(1) $$\sigma_{ij}(r,\theta )=\frac{K}{\sqrt{2\pi \cdot r}}f_{ij}(\theta )+T\delta_{1i}\delta_{1j}$$

where (*r*, *θ*) are polar coordinates with the origin located at the crack tip. *K* is the stress intensity factor; *f_ij_* (*θ*) are the non-dimensional angular functions; *T* is a uniform stress parallel to the crack face, commonly referred to as *T*-stress. *δ*_1*i*_ and *δ*_1*j*_ are Kronecker deltas; indices *i*, *j* have a range from 1–2. The above expression is considered as the *K-T* two-parameter approach for linear elastic material.

Through extending the two-parameter *K-T* linear fracture mechanics (LEFM) approach for elastic material to elastic-plastic material and elastic-plastic fracture mechanics (EPFM), Betegon and Hancock [11], as well as Al-Ani and Hancock [12], developed the *J-T* two-parameter approach. The *J*-integral is used for replacing *K* to characterize the loading level, while *T*-stress is kept as the parameter describing constraint effect. With *T*-stress as an LEFM parameter, the *J-T* approach is only appropriate for SSY condition.

Nikishkov et al. [16,17] suggested *J-A* two-parameter EPFM approach based on *J-A*_2_ approach of Yang et al. [15]. It is presented by a three-term asymptotic expansion but only with two fracture parameters: *J* and *A*, where *A* is the constraint parameter. The three-term asymptotic expression of the *J-A* two-parameter approach for elastic-plastic near-tip stress fields could be presented as (when hardening exponent *n* ≥ 3):

(2) $$\frac{\sigma_{ij}}{\sigma_{0}}=A_{0}{\bar{r}}^{s}{\bar{\sigma }}_{ij}^{\left(0\right)}(\theta )-A{\bar{r}}^{h}{\bar{\sigma }}_{ij}^{\left(1\right)}(\theta )+\frac{A^{2}}{A_{0}}{\bar{r}}^{2h-s}{\bar{\sigma }}_{ij}^{\left(2\right)}(\theta )$$

where power *h* is an eigenvalue depending on hardening exponent *n*; power *s* = −1/(*n* + 1); dimensionless radius $\bar{r}$ is defined as $\bar{r}=r/(J/\sigma_{0})$; *σ*_0_ is yield stress; *σ_ij_* (*θ*) are stress components *σ_r_*, *σ_θ_* or *σ_rθ_* in the polar coordinate system with origin at the crack tip; ${\bar{\sigma }}_{ij}^{\left(0\right)}(\theta )$, ${\bar{\sigma }}_{ij}^{\left(1\right)}(\theta )$ and ${\bar{\sigma }}_{ij}^{\left(2\right)}(\theta )$ are normalized angular functions; the amplitude *A*_0_ = (*σε*_0_*I_n_*) − 1/(*n* + 1). *I_n_* is a scaling integral only depending on material hardening exponent *n*, see [8,9] for details. A computational algorithm developed by Nikishkov [16] could be utilized to determine the values of scaling integral *I_n_*, asymptotic power *h* and normalized angular functions ${\bar{\sigma }}_{ij}^{\left(0\right)}(\theta )$, ${\bar{\sigma }}_{ij}^{\left(1\right)}(\theta )$ and ${\bar{\sigma }}_{ij}^{\left(2\right)}(\theta )$.

With the value determination of *J*-integral and constraint parameter *A*, Equation (2) could be used to characterize crack-tip stress fields of cracked structures. Both analytical and numerical methods for *J*-integral determining are available in literature. The methods to obtain parameter *A* values are quite scarce. In the following paragraphs, an estimate method proposed by the authors [21] will be presented, which directly predicts the parameter *A* values from *T*-stress conveniently and effectively.

### 2.2. T-Stress-Based Estimate of Constraint Parameter A

Under a low load (SSY) condition, the existence of a one-to-one relationship between constraint parameter *A* and *T*-stress was proven analytically by the authors [21]:

(3) $$A=A_{T}\left(\frac{T}{\sigma_{0}},n\right)$$

where symbol *A_T_* implies constraint parameter *A* solutions, which are determined directly from available *T*-stress values.

Based on parameter *A* numerical solutions from FEA results of modified boundary layer (MBL) problem (see Section 3 for details), through least-square fitting, detailed expression of the *A-T* relationship (Equation (3)) could be obtained as three order polynomials for various hardening exponent *n* [21]:

(4) $$A_{T}\left(\frac{T}{\sigma_{0}},n\right)=A_{SSY}(n)+m_{1}(n)\left(\frac{T}{\sigma_{0}}\right)+m_{2}(n){\left(\frac{T}{\sigma_{0}}\right)}^{2}+m_{3}(n){\left(\frac{T}{\sigma_{0}}\right)}^{3}$$

The coefficients *m*_1_(*n*), *m*_2_(*n*) and *m*_3_(*n*) in the Equation (4) polynomials are functions of hardening exponent *n*, whose values are determined in the least-square fitting process. The parameter *A* solution under zero *T*-stress (standard SSY) condition, *A_SSY_*(*n*), is utilized here to closely approximate the *A* values under zero load condition. 

The *T*-stress normalized by yield stress *σ*_0_ in Equation (4), *T/σ*_0_, could be rewritten as the following [21], with external load ratio *σ*/*σ*_0_:

(5) $$\frac{T}{\sigma_{0}}=\frac{T}{\sigma }\cdot \frac{\sigma }{\sigma_{0}}=V\left(\frac{a}{W}\right)\cdot \frac{\sigma }{\sigma_{0}}$$

where *V* is the normalized *T*-stress by external load *σ*, *V = T/σ*, *W* is the specimen width and *a* is specimen crack length. 

Submitting Equation (5) into (4), a detailed expression of *A-T* relationship with external loading ratio *σ*/*σ*_0_ could be obtained [23]:

(6) $$A_{T}\left(\frac{\sigma }{\sigma_{0}},\frac{a}{W},n\right)=A_{SSY}(n)+\left(\frac{\sigma }{\sigma_{0}}\right)g_{1}(\frac{a}{W},n)+{\left(\frac{\sigma }{\sigma_{0}}\right)}^{2}g_{2}(\frac{a}{W},n)+{\left(\frac{\sigma }{\sigma_{0}}\right)}^{3}g_{3}(\frac{a}{W},n)$$

where *g_i_* (*a/W*, *n*) = [*V*(*a/W*)]*^i^ m_i_* (*n*), *i* = 1, 2, 3. With solution determination of normalized *T*-stress (*V*) and the polynomial coefficients *m*_1_(*n*), *m*_2_(*n*) and *m*_3_(*n*), Equation (6) can be used to conveniently determine constraint parameter *A* under SSY for various cracked structures, for example the sensor supporting bracket.

### 2.3. Three-Dimensional (3D) Aspect of Crack Structures

The *T*-stress-based estimate method for constraint parameter *A* solution were developed based on a 2D plane strain condition [21]. However, cracks in practical engineering structures are usually 3D, whose stress (strain) status varies along the 3D crack front and which generally deviates from 2D plane conditions.

Shih et al. [24] argued that when radial radius *r* approaches zero, plane strain conditions should still be dominant. Kim et al. [25] and Zhu et al. [26] found two phenomena in the planes perpendicular to the 3D model crack front (in-plane): (1) stress fields at near crack-front areas are in a plane strain state, while far areas are dominated by plane stress state; (2) through almost the whole thickness of the 3D structure model, in-plane stress fields at the crack front possess plane strain nature, expect the region near the free surface where plane stress status is dominant.

Usually, elastic-plastic fracture mechanics (EPFM) analysis focuses on the crack-tip (-front) region. In addition, crack (fracture) generally occurs in the middle planes of 3D structure. Therefore, the *T*-stress-based estimate method could be applied to a 3D structure through utilizing it in arbitrary planes perpendicular to 3D crack front, except those near a free surface. 

## 3. FEA and Parameter *a* Numerical Solution for 3D Structures under Biaxial Loading

### 3.1. Material Model and Properties

The material model for all finite element analyses (FEA) of current 3D models is the deformation plasticity theory. The Ramberg-Osgood power-law strain hardening relation for 3D cases is available in the commercial finite element code ABAQUS [27]. Following the deformation plasticity theory, the Ramberg-Osgood relation for multi-axial states could be written as:

(7) $$\frac{\varepsilon_{ij}}{\varepsilon_{0}}=(1+\nu )\frac{\sigma_{ij}}{\sigma_{0}}-\nu \frac{\sigma_{kk}}{\sigma_{0}}\delta_{ij}+\frac{3}{2}\alpha {\left(\frac{\sigma_{e}}{\sigma_{0}}\right)}^{n-1}\frac{s_{ij}}{\sigma_{0}}$$

where *σ_ij_* refers to the stress components, strain components *ε_ij_* =*σ_ij_*/*E*, yield strain *ε*_0_ = *σ*_0_/*E*, *σ*_0_ the material yield stress, *α* is a material coefficient, and the material hardening exponent *n* >1. *δ_ij_* is the Kronecker delta, $\sigma_{e}=\sqrt{3s_{ij}s_{ij}/2}$ the von Mises effective stress, and *S_ij_* refers to the deviatoric stress components. 

For the implementation of a 3D FEA, material properties are chosen as follows: elasticity modulus *E* = 2.0 × 10^11^ Pa, yield stress *σ*_0_ = 4.0 × 10^8^ Pa, Poisson ratio *ν* = 0.3, material coefficient *α* = 1.0 and hardening exponent *n* = 3, 5, 10 (3, 4, 5, 7 and 10 for MBL formulation). The material properties chosen cover a wide range of both high and low material strain hardening behaviors.

### 3.2. 3D Modified Boundary Layer Simulation

**Figure 1:**
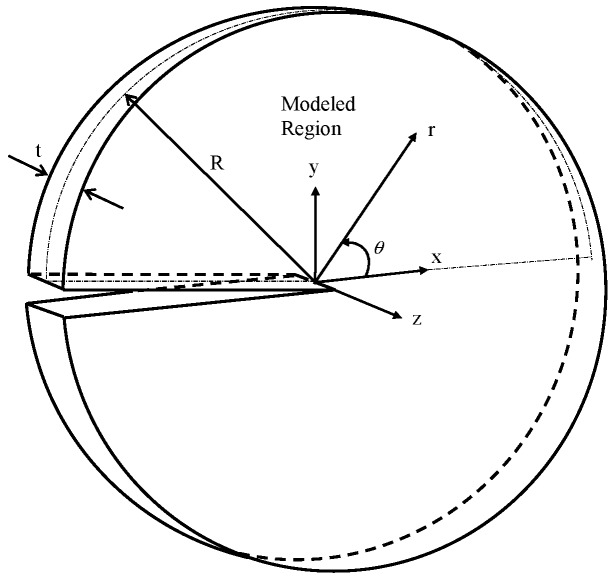
Three-dimensional (3D) modified boundary layer model.

To characterize the small scale yielding (SSY) condition of the 2D or 3D sensor cracked structures, the modified boundary layer (MBL) problem (simulation) could be a practical investigating application that combines the *J-T* and *K-T* two-parameter approaches. Figure 1 illustrates a 3D MBL formulation model, where *R* is the maximum radius, and *t* the model thickness. 

The MBL formulation is a 2D or 3D elastic–plastic near crack-tip (or -front) problem with elastic boundary conditions, i.e. an asymptotic stress field characterized by far-field stress intensity factor *K* and far-field *T*-stress (refer Figure 1). Loadings are applied through displacement boundary conditions, which are expressed by stress intensity factor *K* and *T*-stress. For 3D cases, the applied loadings are set uniformly at the far-field boundary (*r_max_* = *R*) of the MBL model across the model thickness. 

For the 3D MBL model with a small thickness *t*, the formulation could be implemented following the 2D plane stress condition. Displacement components *u_x_* and *u_y_* of the 2D plane stress MBL problem could be determined based on the *K-T* stress fields as [10]:

(8a) $$u_{x}=\frac{K}{2\mu }\sqrt{\frac{r}{2\pi }}\cos\left(\frac{\theta }{2}\right)\left[\kappa -1+2\sin^{2}\left(\frac{\theta }{2}\right)\right]+\frac{1}{2\mu (1+\nu )}Tr\cos\theta$$

(8b) $$u_{y}=\frac{K}{2\mu }\sqrt{\frac{r}{2\pi }}\sin\left(\frac{\theta }{2}\right)\left[\kappa +1-2\cos^{2}\left(\frac{\theta }{2}\right)\right]+\frac{\left(-\nu \right)}{2\mu (1+\nu )}Tr\sin\theta$$

where *μ* is the shear modulus and *ν* is Poisson’s ratio; *κ* = (3 − *ν*)/(1 + *ν*). The far-field stress intensity factor *K* could be determined from the far-field *J*-integral. For the 2D plane stress condition:

(9) $$K=\sqrt{JE}$$

where *E* is the Young’s modulus. To more accurately simulate the remote boundary condition of plane stress MBL model, besides the in-plane displacement components *u_x_* and *u_y_*, out-of-plane displacement component *u_z_* on the boundary should be applied across the model thickness linearly; see reference [28] for an example. 

Kim et al. [25] proved that, in the region near the crack front of a thin 3D MBL model, the FEA results are nearly identical with and without the *u_z_* application on model boundary. The difference only appears in the region near the far field boundary. The current work focused on the crack front region of 3D model. Thus, the *u_z_* application is not included in the FEA process of current thin 3D MBL formulation.

### 3.3. Finite Element Analysis and Numerical Solutions

**Figure 2:**
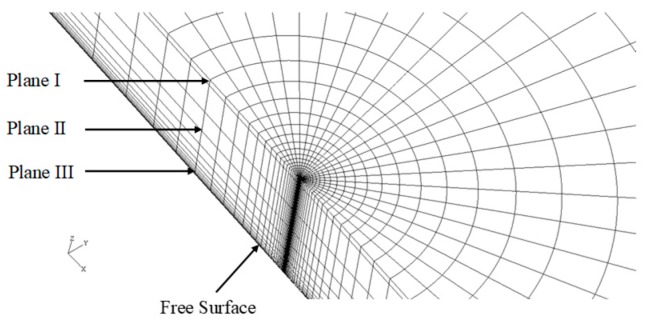
Finite element mesh for 3D modified boundary layer (MBL) model (crack front).

**Figure 3:**
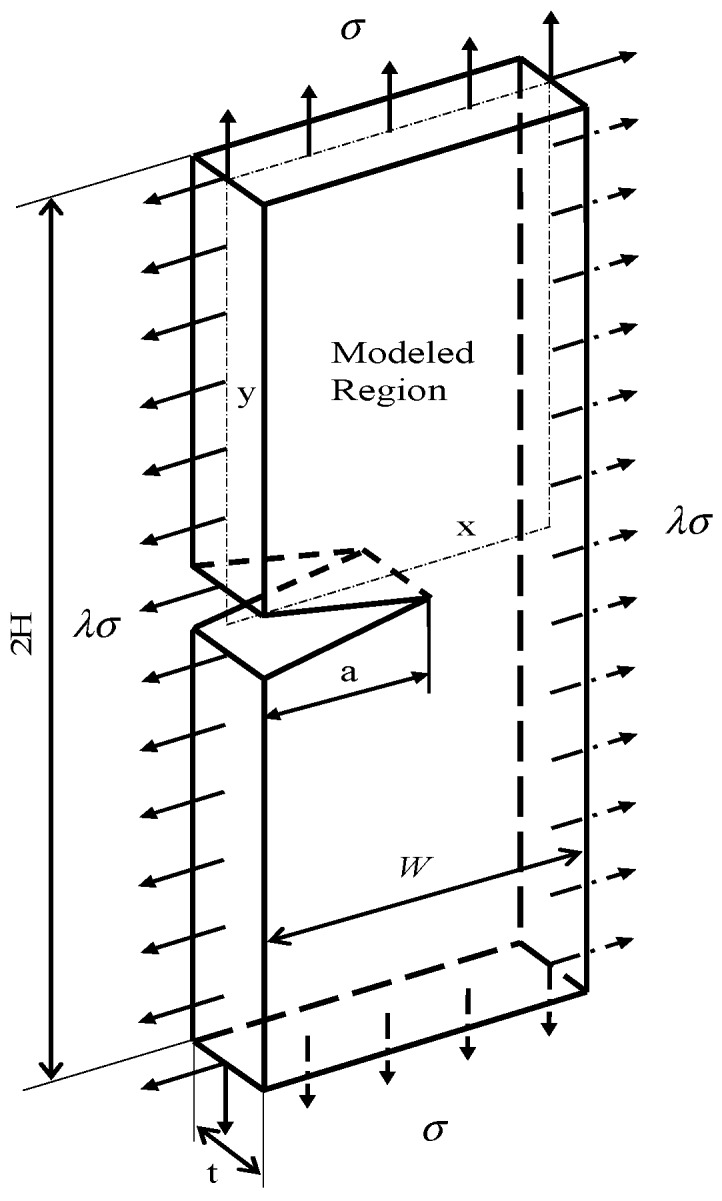
3D single edge cracked plate (SECP) specimen model.

**Figure 4:**
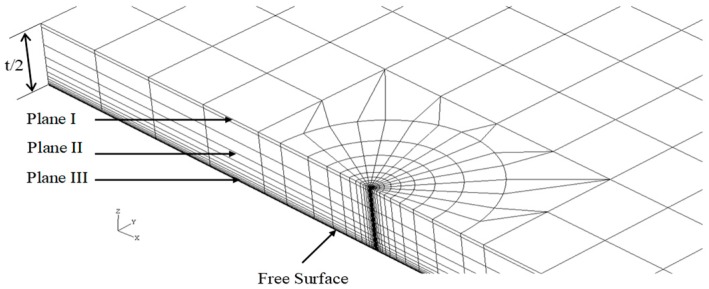
Finite element mesh for 3D SECP specimen (crack front).

Figure 1 illustrates the 3D MBL model, and Figure 2 shows the crack front portion of its FEA mesh. Here, the model thickness *t* was chosen to be 0.04064 m, while the ratio *t*/*R* was fixed at 0.1. The geometry of 3D SECP model was defined by the ratio of height to width, *H/W*, relative crack length, *a*/*W*, and the ratio of model thickness to width, *t*/*W*, see Figure 3. For the current 3D SECP, ratio *H*/*W* was fixed at 3.0 and the ratio *t*/*W* at 0.1, with a set thickness of *t* = 0.04064 m. The FEA mesh crack front portion of 3D SECP is shown in Figure 4. Several various relative crack lengths, *a/W* = 0.1, 0.3, 0.7, were investigated for 3D SECP models. Here, *a/W* = 0.1 describes the shallowest crack depth, *a/W* = 0.3 illustrates a typical medium (average) crack depth among the shallow cracks and *a/W* = 0.7 represents a typical medium (average) crack depth among the deep ones, which typically cover the whole crack depth range from the shallowest to the deepest ones.

Because of the geometrical symmetry, for both MBL and SECP, only one-quarter of the 3D model is meshed for FEA, with coordinates the origin set at the crack tip in the middle plane of the 3D model. The FEA elements near crack-tip (-front) were set around the crack-tip (-front) circularly. Element radial sizes of finite element mesh were varied according to the geometric progression, with *r*_1_ as the element radius of the first circular layer. An inner boundary to the first circular element layer was introduced at *r* = 10^−3^*r*_1_, which was used to simulate the singularity in FEA.

As mentioned before, to analyze 3D models, a couple of typical planes along 3D model thickness (crack front) needed to be chosen for FEA implementation and subsequent structure analysis. As for current 3D MBL and SECP models, planes were chosen as: plane I, *z/t* = 1.4%; plane II, *z/t* = 27.1%, refer Figure 1, Figure 2, Figure 3 and Figure 4. In Figure 3 and Figure 4, the plane III (*z/t* = 49.8%) illustrates a plane near the free surface of 3D model.

For current 3D MBL simulation, the value of far-field *J*-integral was fixed at 2.55 × 10^4^ *J*/*m*^2^. It was related with *K* by Equation (9) to simulate the SSY (low load) condition. Finite element analyses were carried out for various *T*-stress values, *T*/*σ*_0_ = −1.0, −0.8, −0.6, −0.4, −0.2, 0.0, 0.2, 0.4, 0.6, 0.8, 1.0 for several hardening exponent values, *n* = 3, 4, 5, 7, 10, respectively. 

For 3D SECP structures, biaxial loading was applied to the four edges of 3D models, with a biaxial loading ratio *λ* = 1.0.

During the finite element analysis (FEA) process, the plastic zone size was kept smaller than 10% of the in-plane dimensions (*R* for MBL, *W* for SECP). Maximum several plastic zone sizes were approximately 8.6%. It ensured that all the cases of FEA did not exceed SSY range. 

Based on the obtained FEA results, the *J*-integral values could be determined using the domain integral method [29], which is included in code ABAQUS [27]. For constraint parameter *A*, numerical solutions for the 3D structure could be obtained through a fitting method proposed by Nikishkov et al. [16,17], based on the FEA results. See reference [16] and [17] for more details on the fitting method and procedure of determining the *A* values from the FEA results. 

**Figure 5:**
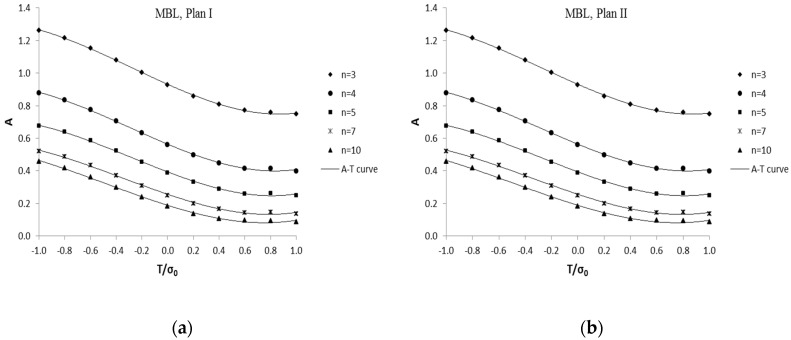
*A-T* relationship curves from 3D MBL formulation, for plane I (**a**) and II (**b**), *λ* = 1.0.

With the obtained FEA results, constraint parameter *A* numerical solutions and *J*-integral values of 3D MBL were obtained for various *T*/*σ*_0_ values [22,30]. Parameter *A* numerical solutions are shown in Figure 5 for plane I and II, and tabulated in Table 1 for Plane II, respectively.

Similarly, numerical solutions of constraint parameter *A* for 3D SECP models could be determined under an SSY condition through the fitting method [30]. Obtained parameter *A* numerical solutions could be utilized for the implementation and validation of extending the *T*-stress-based estimate method to 3D biaxial loading conditions. In the current investigation, numerical solutions of parameter *A* of 3D SECP models, for external load ratio *σ*/*σ*_0_ in plane I (*z/t* = 1.4%) and plane II (*z/t* = 27.1%), are needed.

## 4. Simplified Format of *T*-Stress-Based Estimate Method under Biaxial Loading

### 4.1. T-stress-Base Estimate for 3D Structures

An SSY method predicting constraint parameter *A* values directly from *T*-stress, the *T*-stress-based method, has been developed by authors [21]. It works well for 2D theoretical models. To enable the methodology to be appropriate for sensor structure analysis and design as well as other engineering applications, in this section, the *T*-stress-based estimate will be extended to 3D structures under the biaxial loading condition.

For current 3D case investigation, two specified planes along model thickness, plane I and II, have been chosen in Section 3 for the extension process. Based on extensive finite element analyses, parameter *A* numerical solutions of 3D MBL models for various *T*-stress values were obtained through the fitting method proposed by Nikishkov et al. [17], which are shown in Figure 5 for plane I and II as well as Table 1 for plane II.

Along the model thickness of 3D sensor structures, the values of constraint parameter *A* depend on planes and their locations. Following the rule, the general expression of *A-T* relationship for 3D structures [22] could be derived from Equation (4) as:

(10) $$A_{T}\left(\frac{T}{\sigma_{0}},n,z\right)=A_{SSY}(n,z)+m_{1}(n,z)\left(\frac{T}{\sigma_{0}}\right)+m_{2}(n,z){\left(\frac{T}{\sigma_{0}}\right)}^{2}+m_{3}(n,z){\left(\frac{T}{\sigma_{0}}\right)}^{3}$$

where *z* illustrates the coordinate location of arbitrary plane along 3D model thickness. The coefficients in Equation (10), *m*_1_ (*n*, *z*), *m*_2_ (*n*, *z*) and *m*_3_ (*n*, *z*), depend on hardening exponent *n* and vary along the 3D structure thickness (*z*). 

The values of coefficients *m_i_* could be obtained through the least square fitting procedure based on the numerical solution of parameter *A*. In plane I and plane II, the two specified planes investigated in current work, the values of *m_i_* are determined based on *A* numerical solution of 3D MBL model (see Figure 5, Table 1). The obtained coefficient values are listed in Table 2 for plane I and II. The *A-T* relation curves from Equation (10) are presented in Figure 5 for plane I and II.

Similarly, the detailed expression of *A-T* relationship for 3D sensor structure application could be derived from Equation (6), the external loading of 3D structures is normalized by yield stress *σ*_0_, *σ*/*σ*_0_:

(11) $$A_{T}\left(\frac{\sigma }{\sigma_{0}},\frac{a}{W},n,z\right)=A_{SSY}(n,z)+\left(\frac{\sigma }{\sigma_{0}}\right)g_{1}(\frac{a}{W},n,z)+{\left(\frac{\sigma }{\sigma_{0}}\right)}^{2}g_{2}(\frac{a}{W},n,z)+{\left(\frac{\sigma }{\sigma_{0}}\right)}^{3}g_{3}(\frac{a}{W},n,z)$$

where *g_i_* (*a/W*, *n*, *z*) = [*V*(*a/W*, *z*)]*^i^* *m_i_* (*n*, *z*), *i* = 1, 2, 3. 

### 4.2. Determining Constraint Parameter A for 3D SECP under Biaxial Loading

Under an SSY (low load) condition, through Equation (11), constraint parameter *A* solutions of 3D SECP structure could be obtained directly from *T*-stress. In order to implement *A* value estimate using Equation (11), one has to first determine the values of *A_SSY_* (*n*), coefficients *m_i_*(*n*) and normalized *T*-stress, *V* (*V* = *T/σ*).

The *A_SSY_* (*n*) solutions are already available, see the *A_SSY_* term in Table 2 or *T*/*σ*_0_ = 0.0 case in Table 1. Values of coefficients *m_i_* (*n*) have also been obtained for 3D SECP analysis, see Table 2. 

For 3D structures, Nakamura and Parks [31] developed a numerically computational method for *T*-stress solution along 3D crack fronts (model thickness), which has been adopted in commercial finite element code ABAQUS [27]. Using ABAQUS [27], the proposed numerical method is utilized for current 3D SECP investigation, to compute the *T*-stress (then normalized *T*-stress, *V* (*V = T/σ*)) in the specified planes of 3D SECP. With relative crack length *a*/*W* = 0.1, 0.3 and 0.7, determined *V* (*z*) values for plane I and II are given in Table 3.

### 4.3. Validation and Discuss

Through the *T*-stress-based estimate method for 3D structure, Equation (11), constraint parameter *A* solutions for 3D SECP were obtained conveniently and quickly. In the specified planes of 3D SECP, predicted *A* solutions of 3D SECP were compared with their numerical solutions determined from FEA results. With hardening exponent *n* = 3, 5 and 10, respectively, a comparison was implemented for relative cracks depth *a*/*W* = 0.1, 0.3, 0.7 for chosen planes I and II. 

For 3D SECP, the maximum predicting loads of the developed estimate method, *σ*/*σ*_0_, are shown in Table 4. Within the prediction ranges, i.e. applied external loading less than maximum predicting loads, for both plane I and II, most of the comparing differences were less than 10%; meanwhile, around 35 percent of the differences were less than 5%. Among all of the compared cases, maximum several differences appear as approximately 12% for maximum several external loading applied. Generally, it could be said that good agreement was found for all compared cases. 

**Figure 6:**
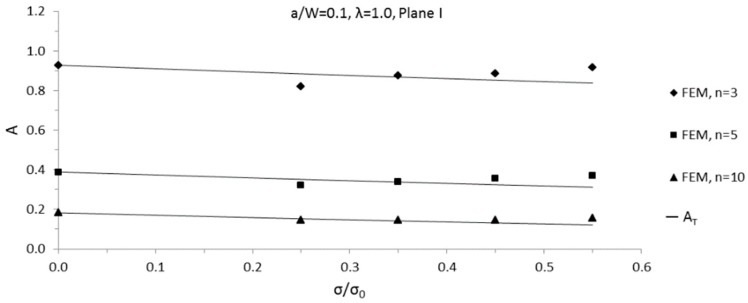
Comparisons of predicted 3D SECP *A* values with finite element analysis (FEA) data, biaxial ratio *λ* = 1.0, *a/W* = 0.1, in plane I.

**Figure 7:**
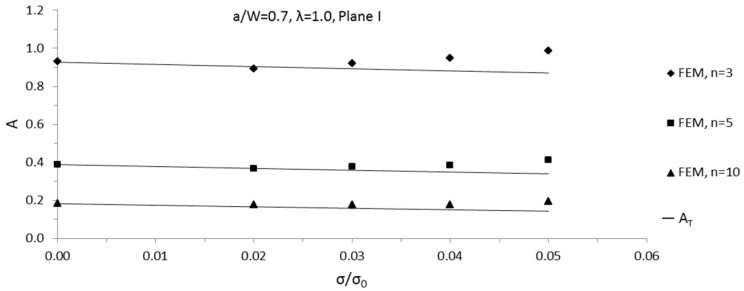
Comparisons of predicted 3D SECP *A* values with FEA data, biaxial ratio *λ* = 1.0, *a/W* = 0.7, in plane I.

The comparison results for plane I with *a*/*W* = 0.1 and 0.7 are plotted in Figure 6 and Figure 7 as samples. To show the curve variation trend more clearly, the curves of *A* versus *σ*/*σ*_0_ from both the FEA results and the estimate method are plotted beyond the predicting ranges of developed estimate method in Figure 6 and Figure 7.

Considering the above comparison for wide range of material and geometrical characteristics, it can be concluded that the developed 3D *T*-stress-based estimate method for constraint parameter *A* could be used well under biaxial loading condition of sensor and other structures.

The maximum predicting loads (applicability range) of the *T*-stress-based estimate for 3D SECP under uniaxial tension loading is shown in Table 5, which is available in previous work [22]. Comparing the current maximum predicting loads of estimate method for biaxial loading (*λ* = 1.0, Table 4) with those for uniaxial loading (*λ* = 0.0, Table 5), it was found, for the deep crack case (*a/W* = 0.7) of 3D SECP, that the applicability range of the *A-T* relation, i.e. dominance of the elastic solution, was nearly unaffected by a change of biaxial load ratio (*λ*). However, for shallow cracks (*a/W* = 0.1 or 0.3), the applicability range got smaller with an increase in remote stress biaxiality. This phenomenon coincided with a finding in a previous research (see reference [30]), namely that for shallower cracks, the change of biaxial load ratio *λ* had a greater effect on the crack constraint condition (constraint parameter *A*).

## 5. Conclusions

Under the small scale yielding (SSY) condition of elastic-plastic fracture mechanics (EPFM), for the *J-A* two-parameter approach, an estimate method (*T*-stress-base estimate method) was developed by the authors [21], which predicts the constraint parameter *A* solutions directly from *T*-stress values. With obtaining parameters *A* values conveniently and effectively, this method will enable the extensive theoretical investigation and engineering application of *J-A* two parameter approach. 

Sensor structure analysis and design and other practical engineering applications focus on 3D structure as well as biaxial external loading. The *T*-stress-based estimate method was developed based on a 2D plain strain condition. The authors extended the proposed estimate method to a 3D uniaxial loading condition in [22]. In the current work, it was extended to a much further 3D biaxial loading condition, which is accurate and appropriate for practical sensor engineering applications, such as fracture analysis and design of sensor supporting brackets. 

Utilizing thin 3D single edge cracked plate (SECP) specimens, the method extension and the corresponding validation process were completed. The extension and validation were implemented in a couple of specific planes along the 3D SECP model thickness, perpendicular to the 3D crack front. To validate the extension of the estimate method to the 3D biaxial loading condition, predicted values of constraint parameter *A* were compared extensively with its numerical solution in the planes. To cover extensive material and geometrical characters of 3D structures, the comparison was implemented for material hardening exponents *n* = 3, 5 and 10, with a relative crack length of *a*/*W* = 0.1, 0.3 and 0.7, respectively.

In the current work, the biaxial loading investigation focused on 3D structure (SECP) with a fixed model thickness. In a further work, the effect of 3D structure thickness on the application (e.g. sensor design) of 3D SSY estimate method should be discussed.

## Figures and Tables

**Table 1:** Parameter *A* values from FEA results for 3D MBL formulation, in plane II, biaxial ratio *λ* = 1.0.

*T*/*σ*_0_	*n* = 3	*n* = 4	*n* = 5	*n* = 7	*n* = 10
−1.0	1.2610	0.8772	0.6779	0.5206	0.4574
−0.8	1.2128	0.8322	0.6439	0.4857	0.4174
−0.6	1.1617	0.7760	0.5862	0.4344	0.3632
−0.4	1.0891	0.7099	0.5269	0.3750	0.3018
−0.2	1.0149	0.6405	0.4615	0.3147	0.2431
0.0	0.9435	0.5731	0.4006	0.2602	0.1903
0.2	0.8792	0.5154	0.3495	0.2157	0.1485
0.4	0.8312	0.4713	0.3103	0.1830	0.1206
0.6	0.7974	0.4404	0.2831	0.1610	0.1035
0.8	0.7730	0.4186	0.2647	0.1473	0.0944
1.0	0.7795	0.4299	0.2775	0.1620	0.1117

**Table 2:** Values of coefficients for polynomial *A-T* relationship, plane I and II.

		*n* = 3	*n* = 4	*n* = 5	*n* = 7	*n* = 10
Plane I	*A_SSY_*	0.9277	0.5591	0.3887	0.2508	0.1828
	*m*_1_	−0.3509	−0.3306	−0.3009	−0.2668	−0.2478
	*m*_2_	0.0786	0.0814	0.0753	0.0797	0.0915
	*m*_3_	0.0954	0.0938	0.0904	0.0761	0.0645
Plane II	*A_SSY_*	0.9435	0.5731	0.4006	0.2602	0.1903
	*m*_1_	−0.3385	−0.3138	−0.2863	−0.2557	−0.2414
	*m*_2_	0.0746	0.0779	0.0753	0.0792	0.0927
	*m*_3_	0.0981	0.0895	0.0846	0.0750	0.0673

**Table 3:** Solutions of normalized *T*-stress (*V*) for 3D SECP under biaxial loading, *λ* = 1.0, in plane I and II.

*z/t*	*a/W* = 0.1	*a/W* = 0.3	*a/W* = 0.7
Plane I	−0.4902	−0.4487	2.4003
Plane II	−0.4821	−0.4307	2.4693

**Table 4:** Maximum predicting loads (*σ*/*σ*_0_) for 3D SECP under biaxial loading (*λ* = 1.0), in plane I and II.

*a/W*		Plane I			Plane II	
	*n* = 3	*n* = 5	*n* = 10	*n* = 3	*n* = 5	*n* = 10
0.1	0.550	0.450	0.450	0.550	0.450	0.400
0.3	0.250	0.200	0.200	0.250	0.200	0.200
0.7	0.050	0.040	0.030	0.040	0.035	0.030

**Table 5:** Maximum predicting loads (*σ*/*σ*_0_) for 3D SECP under uniaxial loading (*λ* = 0.0), in plane I and II.

*a/W*		Plane I			Plane II	
	*n* = 3	*n* = 5	*n* = 10	*n* = 3	*n* = 5	*n* = 10
0.1	0.750	0.650	0.650	0.650	0.550	0.550
0.3	0.350	0.250	0.250	0.250	0.250	0.200
0.7	0.050	0.040	0.030	0.040	0.035	0.030
